# Saving more lives on time: Strategic policy implementation and financial inclusion for safe abortion in Indonesia during COVID-19 and beyond

**DOI:** 10.3389/fgwh.2022.901842

**Published:** 2022-09-06

**Authors:** Putri Widi Saraswati

**Affiliations:** ^1^Scientific Team Member, Indonesia Adolescent Health Association, Jakarta, Indonesia; ^2^Julius Centre for Global Health Sciences and Primary Care, Universitair Medisch Centrum Utrecht, Utrecht, Netherlands

**Keywords:** safe abortion, Indonesia, policy, financing, national health insurance, primary care

## Abstract

Abortion is common in Indonesia, with 79% being unsafe. Unsafe abortion is one of the top five causes of maternal deaths globally. Meanwhile, in Indonesia, the maternal mortality rate (MMR) is still high, with up to 30% of it being related to unsafe abortion. In the COVID-19 pandemic, the number of unsafe abortions is expected to increase along with a 15–30% increase in unintended pregnancies. This will add to the number of maternal deaths on top of direct deaths caused by COVID-19. In Indonesia, access to safe abortion is still limited based on grounds. There are also gaps in implementation and in the financing mechanism of legal safe abortion, especially in the era of the national health insurance scheme *(Jaminan Kesehatan Nasional/*JKN). This brief aims to guide how to equitably implement and inclusively finance safe abortion in Indonesia—in and beyond the COVID-19 pandemic—to gain maximum socioeconomic benefit and leave no one behind.

## Introduction

Unsafe abortion is one of the five major contributors to maternal deaths worldwide (1). This burden is especially prominent in low- and middle-income countries (LMICs). The World Health Organization (WHO) stated that while one in three pregnancies globally end with induced abortions, 45% of those abortions are unsafe (2, 3). Among those that are unsafe, 97% happen in developing countries, especially in Asia (3, 4). In Southeast Asia (SEA), it is estimated that 40.4% of abortions are unsafe, and unsafe abortion contributes to 13% of maternal deaths (2, 5).

In Indonesia, abortion is also common. It is estimated in 2015–2019 that 25 induced (including both safe and unsafe) abortions happen per 1,000 Indonesian women of reproductive age (6). It is similar to the abortion rate in Thailand (25 per 1,000), which is lower than that in SEA (43 per 1,000) but higher than that in Singapore (5 per 1,000) (6). However, it is also estimated that up to 79% of abortions among Indonesians were performed using unsafe traditional methods (7). This situation is of high public health concern, considering that the maternal death rate in Indonesia is still high (305 per 100,000 live births in 2015—one of the highest in SEA) (8). It is difficult to know the exact contribution of unsafe abortion to maternal deaths in Indonesia, but it has been estimated to be ~16–30% (9, 10).

Unsafe abortion is defined as “a procedure for terminating a pregnancy carried out either by persons lacking the necessary skills or in an environment that does not conform to minimal medical standards or both (5).” In Indonesia, the rate of unintended pregnancy is 40 per 1,000 women of reproductive age, with 63% of them ending in abortion (6). Women with unintended pregnancies are more likely to resort to unsafe abortion when safe abortion is unavailable or of poor quality (11). In countries with restrictive abortion laws, abortion does not necessarily happen less frequently but is much more likely to be unsafe (2). In fact, providing safe and legal abortion is critical to preventing death and disability caused by unsafe abortion (11). It is also one of the keys to achieving the Sustainable Development Goals (SDG) 2030—especially Goals 3 and 5 about health and gender equity—although the SDGs do not explicitly or straightforwardly mention abortion (12).

On the other hand, the COVID-19 pandemic has been disrupting health systems worldwide with a significant impact on sexual and reproductive healthcare services such as family planning (FP), maternal and newborn healthcare (MNHC), and safe abortion, especially in LMICs—resulting in a significant rise in the number of unintended pregnancies, unsafe abortions, and subsequent maternal deaths on top of the deaths directly caused by COVID-19 (13, 14). Officials from the Indonesian National Population and Family Planning Board (BKKBN) have publicly stated that the board estimated up to 47% decrease in contraceptive use and a 15–30% increase in unintended pregnancy. This means more than 800,000 pregnancies in Indonesia were unintended in the first quarter of 2020 alone (15, 16). Some studies showed a similar decrease in contraceptive use (17, 18). Another cause of unintended pregnancy during the pandemic is sexual violence, which has been reported to be on the rise, mirroring a global trend (19, 20).

The Indonesian government currently only allows safe abortion based on certain legal grounds regulated by the Government Regulation Number 61 Year 2014 about Reproductive Health (PP 61/2014). Those grounds are to preserve the life and health of the mother (medical emergencies), in case of severe fetal anomalies and in case of rape. It also regulates how safe abortion should be provided. An eligibility team consisting of at least two health professionals (one of them must be a trained medical doctor) should decide on the client's eligibility in the case of a medical emergency. In the case of rape, a legal expert, psychologist, or other experts should confirm there is indeed a suspicion of rape (21). An additional regulation, the Minister of Health's Regulation Number 3 of 2016 (Permenkes 3/2016), regulates that healthcare workers (HCWs) should be trained and organized to provide safe abortion (22).

Furthermore, there is currently no specific policy regulating how to govern, implement, and finance safe abortion in the era of the national health insurance scheme (*Jaminan Kesehatan Nasional* or JKN) in which primary healthcare (PHC) facilities serve as gatekeepers. This is a significant unaddressed gap in safe abortion policy in Indonesia. In the COVID-19 pandemic with limited mobility, access to care, information (including on their legal rights), and fear of infection, Indonesian women who seek abortion may rely even more on informal providers such as online drug sellers who often provide poor-quality service (23).

This brief aims to discuss how strategic policy implementation and inclusive financing can help protect and fulfill the legally mandated right of Indonesian women to access safe abortion to the full extent of the current law, especially in the context of the COVID-19 pandemic, and to mitigate the effect of a future similar health crisis.

## Policy options and considerations

### The role of PHC

The WHO recommends that safe abortion is carried out with the least invasive, most effective, and most appropriate method in relation to pregnancy duration (24). Globally, most abortions occur during the first trimester. Those who get an abortion later are usually due to various barriers in accessing services, especially in LMICs (25–27). A similar trend can be seen in Indonesia (23). The recommended method for abortion during the first trimester is either medical abortion (MA) using a combination of mifepristone and misoprostol, or misoprostol alone when mifepristone is not available, or manual/electric vacuum aspiration (MVA/EVA). A more invasive procedure such as dilatation and curettage (D&C) is no longer recommended. MA and MVA/EVA can be carried out by healthcare providers at the PHC level (24). In various LMIC settings, it has been found that providing safe abortion at the PHC level on an outpatient basis is safe, feasible, and cost-saving (28–30).

#### Policy option 1: Providing safe abortion through an established PHC network

Indonesia has established a wide network of PHC, especially through public facilities (*Puskesmas*), since the 1960 s. Although the private PHC facilities have been available, their roles are significantly expanded after the establishment of the JKN. In the JKN era, PHC facilities (*Fasilitas Kesehatan Tingkat Pertama*, FKTP) play an important role as the first level of contact for individuals, families, and communities, and as gatekeepers (31–34).

PP 61/2014 states that safe abortion can be provided at all levels of care. However, it does not specify any strategic prioritization in terms of access to services. As safe abortion is a lifesaving intervention, it can and should be seen as essential and should be made easily available and accessible. Providing it mainly through FKTP, both public and private, would improve access and responsiveness to the needs of the community. It would also be more cost-efficient than only providing services in secondary care. FKTP is also linked through a geographically established referral system to higher levels of care. This would make referral easier in case of complications.

Another advantage is that by providing it through FKTP, especially *Puskesmas*, safe abortion services can be integrated into the basic MNHC and FP services. For example, clients can be linked directly to post-abortion contraceptive services to prevent unintended pregnancies in future. Clients who do not choose to have an abortion after counseling can also be linked to MNHC services at the same facility. This will improve the continuum of care as a principal of comprehensive MNHC services (35). In addition, this integration can help simplify the mechanism for reporting and purchasing consumables.

There should also be task-shifting between various FKTP providers. There is evidence in LMICs that safe abortion can be provided by HCWs other than medical doctors such as by trained midlevel providers, that is, midwives, nurses, and community health workers (24, 28, 29, 36). The WHO indeed recommends task-shifting to improve cost-efficiency and access to care in providing safe abortion (27). Although PP 61/2014 states that only medical doctors are allowed to perform safe abortions, there are legal possibilities of task delegation between doctors and nurses and/or midwives regulated in Article 65 of the Law of Health Number 36 Year 2009 (UU 36/2009) (37). Furthermore, task-shifting would be especially helpful in the context of human resource scarcity, which is still faced by FKTP in Indonesian rural areas.

This pandemic experience has also brought into light the feasibility of utilizing alternative service delivery modes, like telemedicine, to increase or maintain the accessibility of service and decrease the burden on health facilities on all levels. Telemedicine has been regulated by the Decision of Minister of Health Number HK.01.07/Menkes/4829/2021 (KMK HK.01.07/Menkes/4829/2021) (38). Alongside task-shifting, telemedicine in the case of MA can be used to improve access to safe abortion while empowering clients, giving them more autonomy and dignity (24)—although its use and scale-up would have to take into consideration the differences in infrastructure and network availability across the nation's geography.

There are some implications and disadvantages of this policy option. Technical guidelines and training should be established for safe abortion, especially for FKTP. Resources should be made available for purchasing and stocking by FKTP, such as medications and vacuum aspirators. Misoprostol is not currently included in the latest National Essential Medicine List of 2019, although it is included in the 2019 WHO Model List of Essential Medicines (39, 40). It is only registered for gastric ulcers despite being used frequently for other obstetric indications. Mifepristone is currently not registered in Indonesia (41). Vacuum aspirators are not readily available for FKTP and tend to be used only by specialists or hospitals.

Safe abortion indications stated in the current regulation (PP 61/2014) should also be interpreted broadly to encompass health problems beyond life-threatening conditions. This is to enable FKTP providers to diagnose broader health-related indications according to their level of competence. A broad interpretation of the current safe abortion regulation is crucial to delivering safe abortion to the full extent of the current law and is also in line with the protection of women's right to be free from physical and mental suffering, as stated in the International Covenant on Civil and Political Rights (CCPR) that Indonesia has ratified in 2006 (42, 43). Meanwhile, removing based-on-grounds safe abortion regulation is recommended to fully realize access to safe and legal abortion for all Indonesians, and its advocacy should be continuously advanced (24).

Another possible disadvantage is sociocultural barriers and/or conscientious objections by FKTP providers. To address this, guidelines and training should include value clarification to address HCWs' views on abortion. Safe abortion-specific value clarification work should be based on internationally recognized toolkits (44). Awareness and information should be continuously dispersed among HCWs and health facility managers. Furthermore, an obligation to refer should be explicitly established in the case of conscientious objection to providing abortion. There is currently no specific regulation or policy regarding conscientious objection by HCWs to provide medical treatments in Indonesia. In general, doctors are only legally allowed to refuse clients based on a lack of competence or facilities. In both cases, there is an obligation to refer as well (45). In terms of ethics, there is a potential source of objection based on the Indonesian version of the Hippocratic Oath, which says “to respect all lives starting from the conception,” seen as a part of the “do no harm” principle (46). Regarding this ethical debate, it should be strongly argued that providing services that prevent illness, disability, and death—such as safe abortion—is both highly ethical and countable as “do no harm”.

#### Policy option 2: Providing safe abortion services through contracting the nonprofit private sector

The nonprofit private sector in Indonesia have significant experiences in providing safe abortion-related services in the effort to fill the unmet need. These experiences include care such as pre-and post-procedure counseling, medical service provision, and remote-based services. They have developed technical guidelines according to the WHO guideline and trained providers. They also have extensive experiences in safe abortion advocacy in Indonesia and are aware of the policy development (47–49). Considering their position, resources, and capabilities, the Indonesian Ministry of Health (MoH) could opt to provide safe abortions by contracting them as providers.

This policy option has several advantages. First, there would be less sociocultural resistance from HCWs working with the nonprofit private sector, considering their experiences. Hence, there would be less needs to establish value clarification. Second, their existing guidelines could be adapted as national guidelines and implemented more quickly in the field. Third, the nonprofit private sector already have resources to access tools and consumables related to safe abortion, which would simplify purchasing mechanism.

However, there are also several disadvantages. The nonprofit private sectors only have a limited scale of reach for medical services with limited geographical distribution. This limited distribution can be due to revenue-driven, funder-driven, or urban-preference reasons. Contracting them as sole providers would leave some regions, especially eastern and rural Indonesia, without services. There would also be extra costs related to contract development and administrative or overhead costs. Services provided by the nonprofit private sector are also not yet connected to the public MNCH and FP programs, or the public health program in general. This would separate safe abortion from MNHC and FP programs in general. Separate financing and reporting mechanism would also be needed, which can complicate the governance and monitoring of the services. Additionally, providing an essential service through a third party or outside the public health system might compromise sustainability in the future.

### Financing arrangement

Providing safe abortion is a significant cost-saving approach compared with the cost of unsafe abortion and management of its complication, especially in LMICs. It is estimated that the cost of abortion-related healthcare in LMICs (whose biggest bulk comes from the cost of managing abortion complications) will decrease by up to 78% if all abortions could be provided safely, coupled with expanding access to contraceptive services for women of reproductive age (50). Therefore, public investment in safe abortion services is one of the key steps for a better investment package in sexual and reproductive health in LMICs and for the achievement of universal health coverage (UHC). This is especially urgent considering how the estimated long-term financial shock that the COVID-19 pandemic has brought may affect health financing situation of Indonesia (51).

The Indonesian constitution (UUD'45), the Law Number 40 of 2004 about the National Social Security System (UU 40/2004), and the Law Number 24 of 2011 about the Social Security Administrator (UU 24/2011) have mandated the provision of social security and basic life needs for all Indonesians, including the provision of healthcare services and health security (52–54). Furthermore, protecting Indonesian women from financial risk imposed by the cost of healthcare (including abortion care) is important for the achievement of the UHC (24). As one of the lifesaving and cost-saving interventions in sexual and reproductive health, safe abortion services should be included in the JKN financing scheme.

There are several advantages of this policy option. First, it will improve financial access to services for those who are socioeconomically disadvantaged such as the poor (whose contribution toward the JKN scheme is paid for by the government). Poverty has been known as one of the drivers of poor SRHR outcomes. Poor women and girls in LMICs are at high risk of unintended pregnancy and more likely to be exposed to unsafe abortion (11, 55–57). On the other hand, improving SRHR outcomes and access to services is crucial for poverty reduction. It is crucial that the poor and the marginalized access essential healthcare, including safe abortion (58). Other LMICs such as Nepal and Thailand have made positive impacts by at least partially covering safe abortion costs through public fund (59, 60). Indonesia, with its current 84% coverage for JKN membership, has a chance to do the same (61).

Second, there will be no need to establish a parallel financing arrangement or a separate pooled fund dedicated to safe abortion. This would help make the national pooled funding for health less fragmented as fragmentation leads to less effectiveness and more inefficiency in the system. Reducing fragmentation and aiming for larger revenue pools to increase efficiency are also crucial for the achievement of UHC, including in SRHR (62, 63).

There are several disadvantages and implications of this policy option. The MoH should include tools and consumables related to safe abortion in the National Formulary list of JKN-covered medications, especially for FKTP. There would also be the need to establish a purchasing mechanism for safe abortion in the JKN scheme. This could be done through capitation-based or procedure-related fixed reimbursement for FKTP (as has been done for other basic SRHR services, such as maternal care and long-acting reversible contraceptives/LARC), or through diagnostic-related payment (DRP) for higher levels of care in case of referral (as has been done for specialistic care such as cesarean section). Existing diagnostic lists and reporting mechanisms should also be adjusted to include safe abortion and its indications. In addition, there could be political and/or sociocultural resistance to this policy option as it involves the utilization of public funds. Political engagement, dispersion of information and awareness, and intersectoral/multi-stakeholder dialogue should be actively and continuously carried out to gain support and decrease resistance.

## Recommendation

In line with the policy options that have been presented in this brief, several recommendations are made for the Indonesian MoH and for the national government in general.

### To establish clear technical guidelines for safe abortion as soon as possible

These guidelines should include not only the clinical aspects but also pre- and post-abortion counseling, task-shifting mechanisms, and telemedicine approaches. Guidelines should be developed in line with the WHO's most updated recommendation and informed by best practices in other LMICs.

### To work with professional organizations and the nonprofit private sector to conduct training for HCWs on all levels of care, especially PHC providers, to provide safe abortion

Training should be established as soon as possible on a regular basis and with a proper accreditation mechanism. Training should include clinical aspects, counseling, broad interpretation of the law, and value clarification. Training can be done in partnership with the nonprofit private sector.

### To conduct cost-and-benefit analyses for the integration of safe abortion into the JKN financing scheme, especially for FKTP

Analysis should also include the plan for resource mobilization and allocation.

### To actively engage with HCWs through professional organizations and healthcare facility associations

Information and awareness have to be consistently and continuously dispersed to decrease resistance and improve cooperation among providers. As in training, engagement and advocacy can also be done in partnership with the nonprofit private sector. Engaging with health students and trainees through the curriculum of health education institutions should also be explored to address earlier the ethical consideration and value clarification aspects.

### To link safe abortion services to the national MNHC and FP programs

Linking of the programs should have the provision of a continuum of care and decreasing maternal deaths and disabilities as common goals.

## Conclusion

Providing access to safe abortion services is a crucial public health intervention to decrease maternal deaths and disabilities by driving out unsafe abortion. It is also part of impactful and more cost-efficient investment packages in SRHR, especially in LMICs like Indonesia. As a country that still struggles to decrease its high MMR, Indonesia has taken an important step in establishing several legal grounds for safe abortion services. However, as Indonesia is progressing toward UHC through the establishment of JKN as the national health insurance scheme and amid the challenges brought by the COVID-19 pandemic, policy gaps remain in the form of the uncertainty on how services should be arranged and publicly financed. Making safe abortion services readily available and accessible through the established PHC network and funding the services through the JKN scheme are important to ensure that safe abortion can be equitably delivered to those in need—including the poor, the marginalized, and the most vulnerable—to the full extent of the law and to gain the most socioeconomic benefits for the country.

## Author contributions

The author confirms being the sole contributor of this work and has approved it for publication.

## Conflict of interest

The author declares that the research was conducted in the absence of any commercial or financial relationships that could be construed as a potential conflict of interest.

## Publisher's note

All claims expressed in this article are solely those of the authors and do not necessarily represent those of their affiliated organizations, or those of the publisher, the editors and the reviewers. Any product that may be evaluated in this article, or claim that may be made by its manufacturer, is not guaranteed or endorsed by the publisher.
