# Corrigendum: Saving more lives on time: Strategic policy implementation and financial inclusion for safe abortion in Indonesia during COVID-19 and beyond

**DOI:** 10.3389/fgwh.2023.1129026

**Published:** 2023-01-31

**Authors:** Putri Widi Saraswati

**Affiliations:** Julius Global Health, University Utrecht Medical Center, Utrecht, Netherlands

**Keywords:** safe abortion, Indonesia, policy, financing, national health insurance, primary care

Corrigendum on Saving more lives on time: Strategic policy implementation and financial inclusion for safe abortion in Indonesia during COVID-19 and beyond By Saraswati PW. (2022) Front. Glob. Womens Health. 4:901842. doi: 10.3389/fgwh.2022.901842

**Incorrect References**

In the published article, the reference for Section 2.1.2 Policy option 2, the 1st–5th sentences was incorrectly written as:

47. Perkumpulan Keluarga Berencana Indonesia. Berita Pers PKBI: Penyelamatan Perempuan yang Mengalami KTD Melalui Aturan Layanan Aborsi Aman dan Bertanggung Jawab [Internet]. PKBI. (2015) [cited 2021 Jun 4]. Available from: https://pkbi.or.id/berita-pers-pkbi-penyelamatan467perempuan-yang-mengalami-ktd-melalui-pengaturan-layanan-aborsi-aman-dan-bertanggung468jawab/

48. Safe Abortion Network in Indonesia [470 Internet]. Women on Web. [cited 2021 Jun 4]. Available from: https://www.womenonweb.org/en/page/16338/safe-abortion-network-in-indonesia

49. SAMSARA: My Body My Rights [Internet]. SAMSARA. [cited 2021 Jun 4]. Available from: https://samsara.or.id/

50. Perkumpulan Keluarga Berencana Indonesia. Klinik Kami [Internet]. [cited 2021 Jan 25]. Available from: https://pkbi.or.id/kontak/klinik-kami/

51. Perkumpulan Keluarga Berencana Indonesia. Panduan Aborsi Aman di Klinik PKBI Edisi 2. (2015).

It should be:

47. International Planned Parenthood Federation. IPPF's Annual Performance Report 2007–2008. London, United Kingdom. (2008). Retrieved from: https://www.ippf.org/resource/annual-performance-report-2008-09.

48. Gerdts C, Hudaya I. Quality of care in a safe-abortion hotline in Indonesia: Beyond harm reduction. Am J Public Health. (2016) 106(11):2071–75. doi: 10.2105/ajph.2016.303446

49. Gerdts C, Jayaweera RT, Baum SE, Hudaya I. Second-trimester medication abortion outside the clinic setting: An analysis of electronic client records from a safe abortion hotline in Indonesia. BMJ Sex Reprod Health. (2018) 44(4):286–91. doi: 10.1136/bmjsrh-2018-200102

Hence, references 50 and 51 in the original article shall be deleted, and the total number of references for the article are 63.

The authors apologize for this error and state that this does not change the scientific conclusions of the article in any way. The original article has been updated.

**Text Correction**

In the published article, there was an error. On section 1. Introduction, the 1st sentence of the 3rd paragraph, the word “unintended” should not be there.

A correction has been made to **Section 1 (Introduction),** 3rd Paragraph, 1st sentence. This sentence previously stated:

“Unsafe abortion is defined as ‘a procedure for terminating an unintended pregnancy carried out either by persons lacking the necessary skills or in an environment that does not conform to minimal medical standards or both (5).’”

The corrected sentence appears below:

“Unsafe abortion is defined as ‘a procedure for terminating a pregnancy carried out either by persons lacking the necessary skills or in an environment that does not conform to minimal medical standards or both (5).’”

The authors apologize for this error and state that this does not change the scientific conclusions of the article in any way. The original article has been updated.

**Text Correction**

In the published article, there was an error. On section 2.1.2. Policy option 2, the title of the section, the reference to NGOs shall be reworded.

A correction has been made to **Section 2.1.2**, the section's title. This sentence previously stated:

“Policy option 2: Providing safe abortion services through contracting NGOs.”

The corrected sentence appears below:

“Policy option 2: Providing safe abortion services through contracting the nonprofit private sector.”

The authors apologize for this error and state that this does not change the scientific conclusions of the article in any way. The original article has been updated.

**Text Correction**

In the published article, there was an error. On section 2.1.2. Policy option 2, the 1st paragraph shall be reworded.

A correction has been made to **Section 2.1.2**, the 1st paragraph. This paragraph previously stated:

“Some NGOs in Indonesia have experience in providing safe abortion in the effort to fill the unmet need for this service. Some have their own not-for-profit healthcare facilities, which are mostly outpatient-based, such as clinics and private practices. Some have also had experiences with remote-based or telecounseling. They have developed their own technical guidelines according to the WHO guideline and have their own trained providers. These NGOs have also been actively involved in safe abortion advocacy in Indonesia and are aware of the policy development (47–51). Considering their position, resources, and capabilities, the Indonesian Ministry of Health (MoH) could opt to provide safe abortions by contracting them as providers.”

The corrected paragraph appears below:

“The nonprofit private sector in Indonesia have significant experiences in providing safe abortion-related services in the effort to fill the unmet need. These experiences include care such as pre-and post-procedure counseling, medical service provision, and remote-based services. They have developed technical guidelines according to the WHO guideline and trained providers. They also have extensive experiences in safe abortion advocacy in Indonesia and are aware of the policy development (47–49). Considering their position, resources, and capabilities, the Indonesian Ministry of Health (MoH) could opt to provide safe abortions by contracting them as providers.”

The authors apologize for this error and state that this does not change the scientific conclusions of the article in any way. The original article has been updated.

**Text Correction**

In the published article, there was an error. On section 2.1.2. Policy option 2, 2nd paragraph, the 2nd sentence, the reference to NGOs shall be reworded.

A correction has been made to **Section 2.1.2**, the 2nd paragraph, 2nd sentence. This sentence previously stated:

“First, there would be less sociocultural resistance from HCWs working in these NGOs as they are already experienced as abortion providers.”

The corrected sentence appears below:

“First, there would be less sociocultural resistance from HCWs working with the nonprofit private sector, considering their experiences.”

The authors apologize for this error and state that this does not change the scientific conclusions of the article in any way. The original article has been updated.

**Text Correction**

In the published article, there was an error. On section 2.1.2. Policy option 2, 2nd paragraph, the 6th sentence, the reference to NGOs shall be reworded.

A correction has been made to **Section 2.1.2**, the 2nd paragraph, 5th sentence. This sentence previously stated:

“Third, these NGOs already have resources to access tools and consumables related to safe abortion, which would simplify purchasing mechanism.”

The corrected sentence appears below:

“Third, the nonprofit private sector already have resources to access tools and consumables related to safe abortion, which would simplify purchasing mechanism.”

The authors apologize for this error and state that this does not change the scientific conclusions of the article in any way. The original article has been updated.

**Text Correction**

In the published article, there was an error. On section 2.1.2. Policy option 2, 3rd paragraph, shall be reworded.

A correction has been made to **Section 2.1.2**, the 3rd paragraph. This paragraph previously stated:

“However, there are also several disadvantages. These NGOs only have a limited number of healthcare facilities with limited geographical distribution. For example, Indonesia Planned Parenthood Association (IPPA) only has 23 clinics in 17 of 33 provinces in Indonesia, mostly in western-central Indonesia and urban areas (50). Contracting them as sole providers would leave some regions, especially eastern and rural Indonesia, without services. There would also be extra costs related to contract development and administrative or overhead costs. Some healthcare facilities run by NGOs are already parts of FKTP, but the number is still low (50). This would separate safe abortion from MNHC and FP programs in general. Separate financing and reporting mechanism would also be needed. In addition, providing an essential service through a third party or outside the public health system might compromise sustainability in future.”

The corrected paragraph appears below:

“However, there are also several disadvantages. The nonprofit private sectors only have a limited scale of reach for medical services with limited geographical distribution. This limited distribution can be due to revenue-driven, funder-driven, or urban-preference reasons. Contracting them as sole providers would leave some regions, especially eastern and rural Indonesia, without services. There would also be extra costs related to contract development and administrative or overhead costs. Services provided by the nonprofit private sector are also not yet connected to the public MNCH and FP programs, or the public health program in general. This would separate safe abortion from MNHC and FP programs in general. Separate financing and reporting mechanism would also be needed, which can complicate the governance and monitoring of the services. Additionally, providing an essential service through a third party or outside the public health system might compromise sustainability in the future.”

The authors apologize for this error and state that this does not change the scientific conclusions of the article in any way. The original article has been updated.

**Text Correction**

In the published article, there was an error. On section 3.2. the title, the reference to NGOs and CSOs shall be reworded.

A correction has been made to **Section 3.2**, the title. This sentence previously stated:

“To work with professional organizations, NGOs, and CSOs to conduct training for HCWs on all levels of care, especially PHC providers, to provide safe abortion.”

The corrected sentence appears below:

“To work with professional organizations and the nonprofit private sector to conduct training for HCWs on all levels of care, especially PHC providers, to provide safe abortion.”

The authors apologize for this error and state that this does not change the scientific conclusions of the article in any way. The original article has been updated.

**Text Correction**

In the published article, there was an error. On section 3.2, the 3rd sentence, the reference to NGOs shall be reworded.

A correction has been made to **Section 3.2**, the 3rd sentence. This sentence previously stated:

“Training can be done in partnership with NGOs.”

The corrected sentence appears below:

“Training can be done in partnership with the nonprofit private sector.”

The authors apologize for this error and state that this does not change the scientific conclusions of the article in any way. The original article has been updated.

**Text Correction**

In the published article, there was an error. On section 3.4, the 2nd sentence, the reference to NGOs shall be reworded.

A correction has been made to **Section 3.4**, the 2nd sentence. This sentence previously stated:

“As in training, engagement and advocacy can also be done in partnership with NGOs.”

The corrected sentence appears below:

“As in training, engagement and advocacy can also be done in partnership with the nonprofit private sector.”

The authors apologize for this error and state that this does not change the scientific conclusions of the article in any way. The original article has been updated.

